# BARRIERS TO MENTAL HEALTH TREATMENT IN POLISH EMPLOYER-BASED HEALTH PLANS: INSURANCE LIMITATIONS AND POLICY GAPS

**DOI:** 10.13075/ijomeh.1896.02683

**Published:** 2025

**Authors:** Agata Olearczyk, Kuba Sękowski, Mateusz Jankowski, Andrzej Silczuk

**Affiliations:** 1 SGH Warsaw School of Economics, Health Innovation Unit, Warsaw, Poland; 2 Centre of Postgraduate Medical Education, Department of Population Health, School of Public Health, Warsaw, Poland; 3 Medical University of Warsaw, Department of Community Psychiatry, Warsaw, Poland

**Keywords:** burnout, health promotion, workplace, health insurance, workforce, mental health

## Abstract

**Objectives:**

The current employment market faces many challenges, both from the employers' perspective and that of employees. One of the key health challenges is employees' mental health. Poland has over 17.6 million of active workforce, but each year the number of sick leaves due to mental disorders and occupational burnout is rising, and so are the costs. This leads to lower incomes, higher utilization of medical services, higher spending on medications, and higher social transfers, impacting the country's economy and individuals' quality of living. This research aims to present the current market practice, gaps, and limitations in the coverage of mental health services available through employee medical plans, as well as to provide epidemiological characteristics of sick leave due to mental health issues in Poland.

**Material and Methods:**

This analysis included general terms and conditions of insurance companies providing employee medical plans (group health insurance) available on their websites. Mental health cover and exclusions of liability were analyzed using 6 different criteria. Epidemiological data on sick leaves (absenteeism) were derived from the annual reports published by the Polish Social Insurance Institution.

**Results:**

All insurance companies introduce strong limitations on mental health cover. The gaps apply to the number of packages that cover consultations with psychologists and psychiatrists, as well as the number and type of visits. Most insurers cover no more than 4 visits with a psychologist and psychiatrist per year, excluding psychotherapy (with 1 exception). The main exclusions of liability include treatment of mental illnesses or behavioral disorders and treatment of addiction. There is no coverage for the costs of medications.

**Conclusions:**

The offer for mental health treatment through employee medical plans is strongly limited and does not cover the actual needs. Employees must seek treatment through the public sector or pay out of pocket for services with limited income during sick leave.

## Highlights

- The study presents mental health services available through employee medical plans.
- These plans exclude people with mental illnesses, behavioral disorders, or addiction.
- Cost of medications are not covered by the employee medical plans.
- The study revealed gaps in mental health care within employee medical plans.

## INTRODUCTION

The existing data and forecasts on mental health show that mental illnesses and disorders are a serious and growing worldwide challenge. Depression and alcohol use disorders are the 2 leading mental health conditions globally, among which depression alone is ranked among the top 20 main causes of disabilities. Depression is affecting around 300 million people worldwide, and the number is still rising. At the same time, it is estimated that <25% of people diagnosed with depression have access to the appropriate healthcare services [1]. Mental health disorders and diseases constitute a significant burden for individuals as well as the population, especially when untreated, accounting for 31% of the global burden of disease (GBD). According to WHO, in 2030, depression will be the leading contributor to the GBD, especially in well-developed countries [2]. This trend is also reflected by statistics in Poland.

The growing number of mental illnesses and disorders affects an increasing number of the working population and creates another challenge to employers and national economies. Although research is very limited, the literature indicates a negative association between stress and employees' performance, especially if stress is perceived as a negative [3]. This matter is complicated by the fact that there are different factors that can contribute to work stress, such as workload, autonomy, sense of influence, meaning, and interpersonal relationships [4]. Poor mental health of employees, which can be caused by negative stress at work, is associated with lost productivity, including increased absenteeism and presenteeism [5]. Although the topic of employee mental health is discussed quite frequently in the business environment – especially since the COVID-19 pandemic – and international scientific literature is available, the number of academic studies in this area in Poland remains limited [6]. There are a few national reports conducted by the private companies specialized in human resources (HR) or the stakeholders. For example, according to the report conducted on the working population in Poland in 2022, almost 53% of respondents felt stressed, approx. 65% felt anxiety, and >60% had a lower mood [7]. The results are particularly visible among office workers, for 61% of the respondents. Only 17% of respondents were physical workers, and 22% were from management [7]. Another research, “Mental condition of Polish employees,” shows that 72% of employees feel constant anxiety and 97% of HR teams notice deterioration of mental health and decrease in employee engagement [8]. The research conducted as part of the campaign Closer to Yourself in 2024 showed that 52% of employees felt emotionally overloaded [9].

Mental health problems related to the work environment generate extremely high costs [10]. According to the WHO, an estimated 12 billion working days are lost every year to depression and anxiety at a cost of 1 trillion USD/year in lost productivity due to poor mental health at work [11]. This affects the country's economy as well as households and individuals, facing lower income (in Poland, an employee receives 80% of a monthly salary during sick leave) and higher costs (cost of consultations with a psychiatrist, psychotherapy, and medications).

The Polish healthcare system is based on the mandatory health insurance [12]. The contribution equals 9% and is deducted from employees' salaries every month. Although >60% of all health spending in Poland comes from the National Health Fund, the amount of direct spending of households is rising, reaching 38.6 million PLN in 2022, higher by 4.4% than in 2021 [13]. Most of them are out-of-pocket and intended for the purchase of medicines [14]. Since 2013, the number of patients purchasing refund antidepressants increased by 83%, with 1.7 million patients in 2023 [14].

There are also indirect expenses on healthcare, transferred through the private health insurance, which is gaining popularity each year – in 2022 it stood for 5.4% of all spending in healthcare [13,14]. In general, mental health services are available within the mandatory health insurance (funded by the National Health Fund), private out-of-pocket expenses, and employer-sponsored medical plans.

Employer-sponsored medical plans are commonly offered as non-wage benefits for workers. According to the data published by the Polish Chamber of Insurance (Polska Izba Ubezpieczeń – PIU), the number of people insured with private medical insurance increased from 0.85 million in the fourth quarter of 2013 to 5.39 million in the fourth quarter of 2024 [15]. A significant percentage of occupationally active people in Poland are covered by the employer-sponsored medical plans [15]. Currently, there is no available research on the mental health treatment coverage with a focus on employees through employer-sponsored medical plans, which is gaining popularity in Poland. Due to the growing burden of mental health issues in Poland [16], a study on mental health treatment in Polish employer-based health plans (additional, private services) may contribute to the ongoing debate on the organization of mental health services and access to these services in Poland. Therefore, due to the urgent need to address employees' mental health, this study aimed to present the current market practice, gaps, and limitations in coverage of mental health services available through employee medical plans, as well as to provide epidemiological characteristics of sick leave due to mental health issues in Poland.

## MATERIAL AND METHODS

### Polish employer-based health plans analysis

This study is a policy analysis based on the official documents available in open access under the Polish law. The authors selected insurance companies offering group employee medical plans by the following criteria:

– type of the offer (group medical insurance),
– standard offer for employers of all sizes (no limitation on headcount),
– type of the insurance company (joint-stock company [spółka akcyjna – SA]).

The list of insurance companies operating in Poland was downloaded from the website of the Polish Financial Supervision Authority (Komisja Nadzoru Finansowego), as of June 30, 2025. Based on the available information on the websites, the authors reviewed which companies offer group medical plans without specific limitations. One company was excluded due to headcount limitations (offer addressed to small and medium-sized enterprises). One mutual insurance company was also excluded due to the specific nature of its legal structure (not a joint-stock company). As a result, 7 insurance companies were selected for the analysis.

Next, an analysis of the general terms and conditions (GT&C) and the scope of packages available on the official websites of insurers was conducted. The authors reviewed the availability of consultations with a psychologist and psychiatrist in each package of each insurer and the exclusions of liability.

### Epidemiological data on sick leaves

Epidemiological data on sick leaves (absenteeism) derived from the annual reports published by the Polish Social Insurance Institution (Zakład Ubezpieczeń Społecznych – ZUS) [17]. Sick leaves due to mental health issues were identified by the International Classification of Diseases 10th Revision (ICD-10), code group “F” [17]. Data on the number of sick absence days, causes of sick leave, the most common causes of sick leaves due to mental health issues defined with the ICD-10 codes, the share of sick leaves due to mental health issues among all sick leaves issued in the given year, as well as the gender distribution of sick leaves due to mental health issues. If available, data for the years 2006–2024 were included in epidemiological analysis.

Data were analyzed with MS Excel (Microsoft, Redmond, USA).

## RESULTS

### Polish employer-based health plans – an analysis of market offer

The selection of the insurance companies was based on the list of active joint-stock companies registered in the in the Polish Financial Supervision Authority, offering group employee medical plans without restrictions to the headcount. The analysis included offers from 7 insurance companies with comparisons of their offer in mental health cover as well as exclusions of liability (Table 1).

**Table 1.** Mental health cover and exclusions of liability in the Polish employee medical plans (insurance market), Poland, 2025

Insurance company	Consultation [n/12 months]			Exclusion of liability (outpatient treatment)	Packages with mental health cover/all [n]	Refund for visits outside insurer's network^a^
	psychologist	psychiatrist	specialists for children			
PZU	4 visits	4 visits	not specified	– treatment of addiction – pre-existing condition – treatment caused by alcohol consumption, use of drugs, intoxicants, and psychotropic substances	2/4 with psychiatrist 1/4 with psychologist	102 PLN
Allianz	4 visits	4 visits	psychiatrist: 4 visits	– treatment of mental illnesses or behavioral disorders – treatment after a suicide attempt	1/5	75 PLN
Compensa	in Assistance 1 visit	not covered in standard offer	not specified	– treatment of mental illnesses or disorders including their consequences – treatment of alcoholism – treatment of addiction – treatment after suicide attempt	2/6	500 PLN for psychologist 75–210 PLN for psychiatrist
INTER Polska	20 visits (including psychotherapy)	not covered	not covered	– treatment caused by mental illnesses, post-traumatic encephalopathy (traumatic brain injuries), neuroses – treatment of alcoholism – treatment of addiction – treatment after suicide attempt	1/7	80 PLN
InterRisk	3 visits	3 visits	psychologist: 3 visits	– treatment of mental illnesses – treatment caused by alcohol consumption, use of drugs, intoxicants, and psychotropic substances – treatment after suicide attempt	1/5	75 PLN for psychologist 85 PLN for psychiatrist
TU Zdrowie	4 visits	4 visits	not specified	– treatment caused by mental defects revealed before the age of 1, caused by genetic diseases, prematurity or perinatal damage – treatment after suicide attempt	1/6	75 PLN
Signal Iduna	2 visits	2 visits	not specified	– treatment of mental illnesses and disorders and their consequences – treatment after suicide attempt – psychotherapy	3/5	no information

a The refund may be higher, depending on the type of the price list. Some insurers introduce different price lists depending on the offer and negotiations.

Access to a psychologist and psychiatrist depends on the type of package. None of the insurers provides those services in all of their options. The proportion of plans including any form of mental health support is generally low, ranging from 1 in 7 (INTER Polska) to 3 in 5 (Signal Iduna), and the least expensive packages do not cover mental health services at all. This suggests that mental health care is still treated as a non-essential add-on, rather than a standard component of employee medical plans. This may also lead to the conclusion that employers with lower budgets cannot provide mental health services for their employees through this type of benefit.

Most of the insurers limit the number of psychologist consultations to 2–4 visits/year (12 months). The exception is INTER Polska, which offers ≤20 visits/year, including psychotherapy, but only in the highest package. All other insurers exclude psychotherapy, providing access only to one-time visits or a few consultations during the year.

The same trend applies to psychiatrist consultations, which are also limited to 2–4 visits/year. Compensa and INTER Polska do not provide access to this specialist at all, at least not in their standard offer. This may be challenging for employees who are on medications and require regular consultations, especially at the beginning of the treatment, when consulting the dose, possible side effects, and changing the drug are common. Some of the insurers specify a separate limit to specialists for children (InterRisk and Allianz).

Next to quantitative limits, there are also several exclusions of liability specified in GT&C. Across all insurance companies, there are broad exclusions of liability due to:

– treatment of addiction and alcoholism,
– mental illnesses and behavioral disorders,
– suicide attempts and their consequences,
– treatment caused by substance use.

Some insurers, such as Allianz, exclude any treatment of mental illness altogether, while others, like TU Zdrowie and INTER Polska, exclude coverage for mental conditions linked to genetic or developmental causes. These exclusions are another limitations that undermine the potential benefits of mental health support, especially for individuals with chronic or severe disorders.

The last limitation refers to the reimbursement for consultations obtained outside the insurer's network. The refund rates according to price lists are typically low, ranging 75–102 PLN, which is far from the actual cost of the private consultations. The price for consultation can start from 200 PLN (psychologist) and 280 PLN (psychiatrist), leaving the employee with a significant gap in the coverage of costs.

### Epidemiology of sick leaves due to mental health issues

Between 2006 and 2024, the number of sick absence days due to mental health issues increased from 9.4 million in 2006 to 30.3 million in 2024 (Table 2). Between 2019 and 2024, the number of sick absence days due to mental health issues increased by half. In all analyzed years, females accounted for over half of sick absence days due to mental health issues (Table 2). Since 2015, >60% of sick absence days due to mental health issues have been among females. Between 2006 and 2024, the share of sick absence days due to mental health issues among all sick absence days over doubled – from 5.2% to 12.6% (Table 2).

**Table 2.** Epidemiology of sick leaves due to mental health issues (International Classification of Diseases 10th Revision, code “F”) based on official data from the Polish Social Insurance Institution [17], Poland, 2006–2024

Year	Sick absence days [n (in million)]	Gender distribution [%]		Share of sick absence days due to mental health issues among all sick absence days [%]
		males	females	
2006	9.4	43.6	56.4	5.2
2007	8.7	41.9	58.1	4.7
2008	9.6	41.6	58.4	4.7
2009	11.4	42.0	58.0	5.3
2010	11.3	41.8	58.2	5.5
2011	12.7	40.6	59.4	6.1
2012	14	40.3	59.7	6.8
2013	15.6	40.5	59.5	7.3
2014	16.1	40.1	59.9	7.6
2015	17.9	38.8	61.2	7.9
2016	19	39.4	60.6	7.9
2017	19.4	39.6	60.4	7.9
2018	19.4	39.5	60.5	8.0
2019	20.2	38.0	62.0	8.5
2020	27.7	36.7	63.3	10.8
2021	25.2	37.2	62.8	10.5
2022	23.8	37.6	62.4	10.0
2023	26.1	37.9	62.1	11.0
2024	30.3	38.2	61.8	12.6

Depressive episode, recurrent depressive disorders, other anxiety disorders, and reaction to severe stress and adjustment disorders were the most common causes of sick absence days due to mental health issues in Poland (Table 3). Since 2012, the share of reaction to severe stress and adjustment disorders among all sick absence days due to mental health issues has increased (Table 3). In 2024, reaction to severe stress and adjustment disorders was the major cause of sick absence days caused by mental health issues, accounting for 35.3% of all sick absence days due to mental health issues. Between 2012 and 2024, the share of depressive episodes and recurrent depressive disorders among the number of sick absence days caused by mental health issues has decreased (Table 3). Between 2012 and 2024, a significant increase in sick absence days caused by other anxiety disorders was reported (Table 3).

**Table 3.** The most common causes of sick absence days due to mental health issues, based on official data from the Polish Social Insurance Institution [17], Poland, 2012–2024

Year	Sick absence days [%]			
	depressive episode (F32)	recurrent depressive disorders (F33)	other anxiety disorders (F41)	reaction to severe stress and adjustment disorders (F43)
2012	24.3	10.5	10.1	23.8
2013	24.1	10.4	10.8	25.6
2014	22.8	10.3	11.8	26.2
2015	21.8	10.2	12.5	27.8
2016	20.8	10.1	13.3	28.6
2017	19.8	10.4	13.7	29.0
2018	18.9	10.2	14.2	30.0
2019	19.5	10.1	15.0	32.5
2020	18.8	9.4	17.8	35.3
2021	19.9	9.2	17.5	34.0
2022	18.0	8.9	16.9	32.9
2023	17.9	8.7	17.5	33.9
2024	17.6	8.5	18.1	35.3

Most common reasons for sick absence days caused by mental health issues – percentage of International Classification of Diseases 10th Revision (ICD-10) diagnoses among all sick leave due to mental health issues.

## DISCUSSION

This is the first study on mental health treatment in Polish employer-based health plans. This study showed differences in mental health coverage and exclusions of liability between the most recognized insurance companies in Poland. Access to a psychologist and psychiatrist is limited and depends on the type of package. Moreover, the currently available employer-based health plans do not address the emerging mental health issues, like reaction to severe stress and adjustment disorders or anxiety disorders, that are major causes of sick absence days caused by mental health issues. Epidemiological data on sick leave among working adults showed that mental health issues are an emerging problem, and the number of sick absence days is rapidly increasing, especially after the COVID-19 pandemic. This study showed that employer-based health plans offer limited mental health services and are not adjusted to the current burden of mental health issues among working adults.

The psychiatric care system within the public healthcare system in Poland is constantly changing [18]. Due to the growing burden of mental health issues in Poland [16], there is a need to increase the capacity of the mental health care system and develop services that would provide mental health treatment. Working adults are a particularly important group to receive care in the event of mental health issues, as absenteeism significantly impacts society and the economy. The data presented in this article show that despite the increasing number of sick days due to mental health issues, employer-based health plans do not provide adequate psychiatric care and provide very limited support for the public mental health care and treatment system.

While discussing the market of private health insurance in Poland, it is important to acknowledge that the Polish market is strongly dominated by subscription contracts, offered by medical companies. There are no legal requirements to publish data on the number of patients with the subscription, so the demographic characteristics of patients covered by the subscription are unknown.

When it comes to the Polish labor market, it is very popular to provide employees with a private medical plan (in the form of an insurance or a subscription). Due to long waiting times in the public sector, employee medical plans became a significant non-wage employee benefit [19]. The current offer of private health insurance is primarily supplementary to the public system, i.e., covering the basket of guaranteed benefits, but giving faster access to services, a wider choice of service providers, and higher quality of services. However, the scope of services for mental health treatment under those is very limited or unavailable. As we can see from the data presented on the rising number of mental health issues among the workforce resulting in absenteeism and its consequences, the coverage of this area is becoming critical.

The effects of long-lasting stress on health are well researched [20]. A common and often cited consequence of excessive stress at work is occupational burnout. Since January 1, 2022, occupational burnout has been officially recognized by WHO in ICD-11 as an “occupational phenomenon,” yet not a condition [21]. However, occupational burnout may lead to other conditions classified as diseases that may result in absenteeism and sick leave at work. Burnout can lead to increased allostatic load, structural and functional brain changes, excitotoxicity, systemic inflammation, immunosuppression, metabolic syndrome, cardiovascular disease, and premature death [22]. A harmful work environment (not only physical but also social) can increase the risk of developing various diseases and disorders [23].

Increasing evidence supports the effectiveness of workplace interventions aimed at protecting and promoting the mental health and well-being of employees [24]. Workplace interventions aimed at preventing mental health issues are expected to show positive effects such as increased self-efficacy, higher person-job-fits, and reduced absenteeism [25] and demonstrate good value for money [26]. Employers' actions may therefore include providing access to dedicated benefits (such as medical care and Employee Assistance Programs) as well as initiatives that promote health. However, the complexity of this area means that employer efforts go beyond strictly health-related products and extend to the broader organizational culture [27]. Research shows that health-promoting leadership (e.g., management) plays an important role in improving employee health and maintaining their well-being [28]. It is also shown that interventions should be addressed to the actual needs of employees, since poorly planned activities may not achieve the desired results [29].

Furthermore, legislative steps have also been taken to motivate employers to take preventative measures. An example is the publication in June 2021 of the new ISO 45003 standard on employee mental health support. This standard, for the first time, introduces a formal framework and guidelines for managing and protecting mental well-being in the workplace [30].

The employer-based medical plans for employees are a popular non-wage benefit in Poland and could be a significant tool for the prevention and treatment of mental health issues, as well as promotion of a healthy work environment. However, when it comes to mental health cover, the authors of this research identified severe gaps in cover and limitations of liability. Employees must seek treatment through the public sector or pay out of pocket for services with limited income during sick leave.

The public healthcare, on the other hand, provides comprehensive treatment, both outpatient and inpatient. However, the challenge is waiting times. According to the Watch Health Care Foundation report [31], in 2022, the waiting time to a mental health clinic was 4.5 months. The waiting time for a consultation with a psychiatrist was 1.9 months, and it increased compared to 2021. In 2025, the highest average waiting times for psychologist and psychiatrist consultations were in the Pomeranian district (220 days and 308 days, respectively) [32].

The consequences of poor mental health among employees affect not only their work performance but also have much broader impacts. It can lead to decreased satisfaction, occupational burnout, and a higher risk of accidents at work [33], as well as absenteeism and presenteeism. Further consequences also impact work colleagues, families, and other members of communities. Last but not least, poor mental health of workers puts at risk the government's budget, social funds, and healthcare costs on many levels. Given the rising incidence of mental illnesses and disorders, the current offer does not cover the actual needs, adding another challenge for employers, employees, and the country's economy. Therefore, developing systemic solutions and standards for protecting employees' mental health becomes critical.

The conducted analysis has some limitations. The research presented was prepared based on the GT&C available on insurers' websites. In terms of group medical plans, it is possible to negotiate terms, adding more visits or higher refund price lists. However, the market practice strongly limits those specialists and does not cover therapy or medications. Data on sick absence days are limited to basic characteristics as published in official reports of ZUS.

## CONCLUSIONS

The conducted analysis shows that the coverage for mental health treatment in employee medical plans is strongly limited and deficient due to the level of package, number of services, type of services, exclusions of liability, and refund rates. There is a lack of comprehensive mental health treatment in employee medical plans that may affect the well-being of workers. Further actions are needed to strengthen mental health services targeted at workers.
